# Oxygen-radical pretreatment promotes cellulose degradation by cellulolytic enzymes

**DOI:** 10.1186/s13068-017-0979-6

**Published:** 2017-12-04

**Authors:** Kiyota Sakai, Saki Kojiya, Junya Kamijo, Yuta Tanaka, Kenta Tanaka, Masahiro Maebayashi, Jun-Seok Oh, Masafumi Ito, Masaru Hori, Motoyuki Shimizu, Masashi Kato

**Affiliations:** 1 Faculty of Agriculture, Meijo University, Nagoya, Aichi 468-8502 Japan; 2 Faculty of Science and Technology, Meijo University, Nagoya, Aichi 468-8502 Japan; 3 Institute of Innovation for Future Society, Nagoya University, Nagoya, Aichi 464-8603 Japan

**Keywords:** Atmospheric pressure plasma, Biorefinery, Cellulose, Oxygen-radical pretreatment, Plant biomass

## Abstract

**Background:**

The efficiency of cellulolytic enzymes is important in industrial biorefinery processes, including biofuel production. Chemical methods, such as alkali pretreatment, have been extensively studied and demonstrated as effective for breaking recalcitrant lignocellulose structures. However, these methods have a detrimental effect on the environment. In addition, utilization of these chemicals requires alkali- or acid-resistant equipment and a neutralization step.

**Results:**

Here, a radical generator based on non-thermal atmospheric pressure plasma technology was developed and tested to determine whether oxygen-radical pretreatment enhances cellulolytic activity. Our results showed that the viscosity of carboxymethyl cellulose (CMC) solutions was reduced in a time-dependent manner by oxygen-radical pretreatment using the radical generator. Compared with non-pretreated CMC, oxygen-radical pretreatment of CMC significantly increased the production of reducing sugars in culture supernatant containing various cellulases from *Phanerochaete chrysosporium*. The production of reducing sugar from oxygen-radical-pretreated CMC by commercially available cellobiohydrolases I and II was 1.7- and 1.6-fold higher, respectively, than those from non-pretreated and oxygen-gas-pretreated CMC. Moreover, the amount of reducing sugar from oxygen-radical-pretreated wheat straw was 1.8-fold larger than those from non-pretreated and oxygen-gas-pretreated wheat straw.

**Conclusions:**

Oxygen-radical pretreatment of CMC and wheat straw enhanced the degradation of cellulose by reducing- and non-reducing-end cellulases in the supernatant of a culture of the white-rot fungus *P. chrysosporium*. These findings indicated that oxygen-radical pretreatment of plant biomass offers great promise for improvements in lignocellulose-deconstruction processes.

**Electronic supplementary material:**

The online version of this article (10.1186/s13068-017-0979-6) contains supplementary material, which is available to authorized users.

## Background

Cellulose is the most abundant polysaccharide found in nature, consists of a β-1,4-linked linear chain of glucose units, and is used in the biofuel, oil, food, textile, and pulp industries [1, 2]. Cellulolytic enzymes are important reagents in industrial biorefinery processes, such as the production of biofuels from plant biomass [3]. The complete degradation of cellulose requires the synergistic action of a set of cellulolytic enzymes with various substrate specificities.

White-rot basidiomycetes are responsible for the complete degradation of lignocelluloses and produce several cellulolytic enzymes, including endo-glucanases (EC 3.2.1.4), cellobiohydrolases (CBHs; EC 3.2.1.91 and EC 3.2.1.176), and β-glucosidases (EC 3.2.1.74) [4, 5]. Endo-glucanases randomly hydrolyze the internal β-1,4-linkage of the glucose backbone, whereas CBHs release cellobiose from both the reducing and non-reducing ends of cellulose polymers [5–8]. CBHI (EC 3.2.1.176) attacks the reducing ends of cellulose polymers, whereas CBHII (EC 3.2.1.91) attacks the non-reducing ends [5–8]. Beta-glucosidase alleviates the inhibitory effect of cellobiose on endo-glucanase activity by hydrolyzing the substrate to glucose [7]. In addition to hydrolytic enzymes, white-rot basidiomycetes produce an extracellular oxidative enzyme in the form of a copper-dependent lytic polysaccharide monooxygenase (LPMO) [9]. LPMOs belong to the auxiliary activity (AA) family of enzymes that includes AA9, AA10, AA11, and AA13 in the CAZy family and is capable of boosting the activity of classical cellulolytic enzymes, leading to a reduced enzyme load for cellulose deconstruction [10–12]. LPMOs combined with cellulolytic enzymes offer an opportunity to improve lignocellulose-deconstruction processes [13, 14].

The ability of LPMOs to boost the activity of classical glycoside hydrolases (GHs) has been among the key drivers for the production of second-generation biofuels. The combination of LPMO belonging to AA9 and cellobiose dehydrogenase results in between two and eightfold improvements in glucose yields from bacterial cellulose, phosphoric acid-swollen cellulose, and microcrystalline cellulose (MCC) when pretreated with several cellulases [15]. In addition, cellulose oxidation by LPMO causes chain cleavage. The boosting effect of LPMO on cellulose degradation is considered a result of generation of new chain ends upon which GHs can act [16, 17]. Igarashi et al. [18] reported that the surface of crystalline cellulose favors the formation of traffic jams of productively bound cellulases. Increasing the number of new chain ends of cellulose polymers by means of pretreatment or the combined use of synergistically acting enzymes should reduce polymer entanglement, thereby improving the mobility of cellulase molecules and increasing hydrolytic efficiency.

Bioethanol production from lignocellulose generally involves three steps: (1) pretreatment to break down the complex lignocellulose structures; (2) enzymatic hydrolysis of polysaccharides (i.e., cellulose and hemicellulose) into fermentable sugars; and (3) fermentation to convert sugars into ethanol [19]. Various biological, chemical, and physical pretreatment methods have been developed [20–27]. In biological pretreatment processes, fungi, such as brown-, white-, and soft-rot basidiomycetes, are used to degrade cellulose, hemicellulose, and lignin, although the rate of degradation is generally low [28]. Chemical methods, such as alkali pretreatment, have been extensively studied and demonstrated as effective for breaking recalcitrant lignocellulose structures; however, these methods have a detrimental effect on the environment. In addition, utilization of these chemicals requires alkali- or acid-resistant equipment and a neutralization step. Recently, a combination of chemical and mechanical pretreatment methods using ozonolysis and ball milling, respectively, improved the enzymatic saccharification of corn straw relative to other pretreatments [29]. The authors clearly showed that the oxidization process using ozone facilitates enzymatic saccharification. To the best of our knowledge, ozone is not a major oxidant, but is a precursor of atomic oxygen.

Our previous work developed radical generators based on non-thermal atmospheric pressure plasma technology [30, 31] using a commercially available radical generator with an oxygen–argon gas mixture to generate oxygen radicals. Furthermore, the use of a large amount of argon provides high electron density on the order of 10^15^ cm^−3^. A previous study reported large amounts of atomic oxygen O (^3^ *P*_*j*_) at an absolute density on the order of between 10^13^ and 10^14^ cm^−3^ (equal to 1–10 ppm) as measured by vacuum ultraviolet absorption spectroscopy (VUVAS) [32]. This oxygen-radical generator was demonstrated to be effective at microorganism sterilization due to oxidization [32, 33]. These results represented a significant optimization of the oxidization process, especially for enzymatic saccharification. Use of the radical generator for pretreatment has several advantages: (1) on-site generation, thereby avoiding problems associated with chemical supply and storage; (2) reaction at ambient temperatures and pressures; and (3) achievement of rapid reaction with a high density of atomic oxygen radicals [VUVAS and UVAS; the densities of atomic oxygen (~ 2.3 × 10^14^ cm^−3^) and O_3_ (~ 2.5 × 10^13^ cm^−3^) equal to ~ 0.94 ppm were measured, respectively] [32]. Furthermore, pretreatment using a radical generator is more environmentally friendly than chemical methods, given that no chemical waste is produced.

In this study, the effects of oxygen-radical pretreatment on enzymatic hydrolysis of carboxymethyl cellulose (CMC) and wheat straw were analyzed. Oxygen-radical pretreatment of CMC and wheat straw enhanced cellulose degradation by reducing- and non-reducing-end cellulases in the supernatant of a culture of the white-rot fungus *Phanerochaete chrysosporium*.

## Methods

### Chemicals and materials

CMC (Hercules, Wilmington, DE, USA) and MCC (Funakoshi, Tokyo, Japan) were used for enzymatic assays. CBHI and CBHII were obtained from Megazyme International (Bray, Ireland). Wheat straw was grown and harvested on the farm of Meijo University (Nagoya, Japan). The straw was cut, dried, and milled to a final particle size of 1 mm, followed by washing at a weight ratio of 1:20 of wheat straw to distilled deionized water. The washed wheat straw was dried and used for oxygen-gas and -radical pretreatment.

### Strains, cultures, and media

*Phanerochaete chrysosporium* (ATCC 34541; ATCC, Manassas, VA, USA) was cultured in liquid Kirk medium [1.2 g/L ammonium tartrate, 0.05 g/L MgSO_4_, 0.01 g/L CaCl_2_, 0.20 g/L K_2_HPO_4_, 1 μg/L thiamine, and 1 mL/L trace mineral solution (pH 4.5)] at 37 °C for 0–14 days and in the presence of 1.0% MCC as the sole carbon source. *P. chrysosporium* was inoculated in 100 mL liquid Kirk medium containing 1.0% MCC as the sole carbon source at 37 °C with shaking at 150 rpm. The culture supernatant was concentrated using an Amicon Ultra filter unit (Merck-Millipore, Billerica, MA, USA) and dialyzed against 50 mM acetate buffer (pH 4.5). All protein-collection steps were performed at 4 °C.

### Proteomic analysis of extracellular proteins produced by *P. chrysosporium*

*Phanerochaete chrysosporium* was cultured in Kirk medium containing 1.0% MCC as a carbon source for 7 days at 37 °C. Extracellular proteins from culture filtrates were separated by two-dimensional electrophoresis (2-DE) and stained with SYPRO Ruby (Bio-Rad Laboratories, Hercules, CA, USA). Protein spots were excised from the gels, digested with trypsin, and analyzed using matrix-assisted laser desorption time-of-flight (TOF)/TOF-mass spectrometry (MS) as previously described [34–36]. Peptide mass fingerprinting (PMF) and tandem MS (MS/MS) spectra were analyzed using the MASCOT search engine (Matrix Science, London, UK) as previously described [34, 35].

### Oxygen-radical pretreatment

A commercially available oxygen-radical generator (Tough Plasma FPA10; Fuji Machine MFG, Aichi, Japan) was used for this study. Briefly, the oxygen-radical generator is based on an atmospheric pressure-discharge plasma operated with a gas mixture containing a small amount of O_2_ (30 sccm) in argon (4.97 slm). The use of large amounts of argon provides a high electron density on the order of 10^15^ cm^−3^ [30, 31]. In addition, it is expected that the use of argon as a buffer decreases the three-body collision between oxygen species resulting in O_2_ and O_3_ molecules, thereby increasing atomic oxygen production in the atmosphere. The structure of the nozzle exit (a slit) with a bent flow channel downstream intercepts high-energy photons, and the electrically grounded potential on the flow channel terminates charged species.

**Fig. 1:**
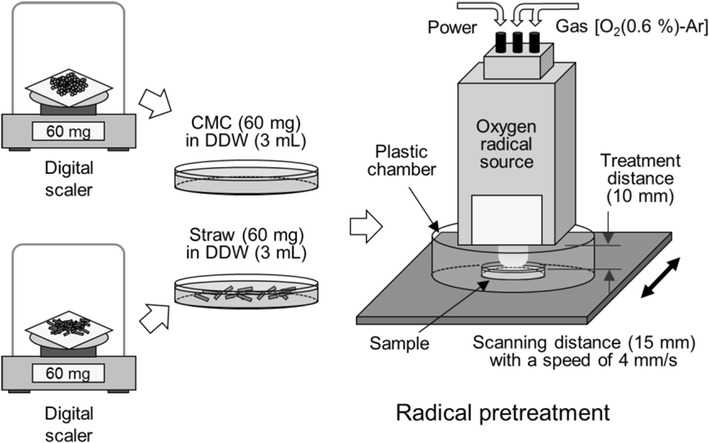
Schematic diagram of sample preparation for oxygen-radical pretreatment. Radical-pretreatment conditions (i.e., mixed gas with 0.6% of O_2_ in argon; treatment distance of 10 mm with a plastic cover) were optimized to obtain maximal atomic oxygen [O (^3^ *P*_*j*_)]

Figure 1 shows a schematic illustration of the preparation of the CMC or wheat-straw aqueous suspension and radical pretreatment using the oxygen-radical source. CMC (60 mg), MCC (60 mg), or wheat straw (60 mg) samples were suspended in 3 mL distilled deionized water, and a fixed distance of 1 cm was used between the slit exit of the radical generator and the surface of the liquid suspension. The suspension samples in Petri dishes (30-mm diameter) were placed on an automated stage for uniform pretreatment of the solution due to the shape of the radical exit (0.5 × 16 mm). The speed of the automated stage was set at 4 mm/s, and a plastic chamber was covered to avoid mixing with ambient air. A pretreatment time of 20 min was used.

### Viscosity measurement of the CMC solution

Viscosity measurements were performed using the aqueous suspension of CMC pretreated with the oxygen radical. The suspension before pretreatment was prepared to allow the CMC concentration to be set to 2.0% (w/v). CMC aqueous suspensions pretreated by blowing the oxygen gas into the suspension were also prepared as mock controls. The pretreatment times were 0, 4, 8, 12, 16, and 20 min for both solutions. Solution viscosity was measured using a rheometer (AR-2000ex; TA Instruments, Tokyo, Japan) with an electrically heated plate and a hard anodized aluminum cone geometry. The cone diameter and angle were 60 mm and 1°1′1″, respectively. Approximately 1.1 mL of the solution was placed onto the heated plate, and the excess solution was removed prior to performing the measurement. The shear rate applied to the samples was 200 s^−1^, and the preliminary measurement was conducted for 2 min to maintain flow conditions. The temperature of the samples was set to 30.0 °C during measurements. The variation in CMC concentration due to water vaporization was reduced using a solvent trap, with water was used as a liquid sealant.

### Enzyme assays

Reducing-sugar production was assayed in 0.5-mL reaction mixtures containing 50 mM acetate buffer (pH 4.5) and culture supernatant, CBHI or CBHII, and 1.0% CMC, MCC, or wheat straw. A 0.1-mL volume of *P. chrysosporium* supernatant grown with MCC for 7 days was added at a final protein concentration of 0.5 mg/mL. Assays with purified CBHI or CBHII were also performed at a protein concentration of 0.3 mg/mL. Reactions were incubated at 37 °C, and the enzymes were removed from the reaction solution using a Nanosep centrifugal device (Pall Corporation, Port Washington, NY, USA) according to the manufacturer’s instructions. Flow-through fractions were boiled at 100 °C for 30 min. After removing the enzymes, the reducing sugars produced were measured using the dinitrosalicylic acid (DNS) method [34, 37, 38]. Standard curves were prepared based on solutions containing different glucose concentrations. One unit of cellulase activity was defined as the amount of enzyme required to produce 1 μmol of reducing sugar (glucose equivalents) per min.

Soluble products released from CMC and wheat straw were determined by monitoring post-column derivatized reducing sugars that were separated using a Prominence reducing-sugar high-performance liquid chromatography (HPLC) analytical system (Shimadzu, Kyoto, Japan) equipped with a fluorescence detector. The supernatant was separated on a Shim-pack 4.0 × 250-mm ISA-07/S2504 column (Shimadzu) with a linear gradient of 0.1 M potassium borate buffer (pH 8.0) and 0.4 M potassium borate buffer (pH 9.0) for 120 min at a flow rate of 0.6 mL/min [34, 37].

### Analytical methods

The composition of cellulose, hemicellulose, and lignin in non-pretreated, oxygen-gas-pretreated, and oxygen-radical-pretreated wheat straw was determined according to previously described methods [39, 40]. Oxygen-gas- and oxygen-radical-pretreated wheat-straw samples were extracted with water to remove inhibitors for enzymatic reactions. Each treatment portion was washed separately at a weight ratio of 1:10 of pretreated wheat-straw samples to 25 °C Milli-Q water. The mixture was stirred at 100 rpm for 60 min, and the extract was filtered through a nylon membrane (pore size: 0.45 μm). The liquid fraction was lyophilized, trimethylsilylated, and analyzed using a GCMS-QP2010 (Shimadzu) equipped with a J & W DB-5MS capillary column (30 m × 0.25 mm internal diameter × 0.25 μm film thickness; Agilent Technologies, Santa Clara, California, USA) [41].

## Results and discussion

### Time-course of cellulase activity and identification of secreted proteins in the culture supernatant

**Fig. 2:**
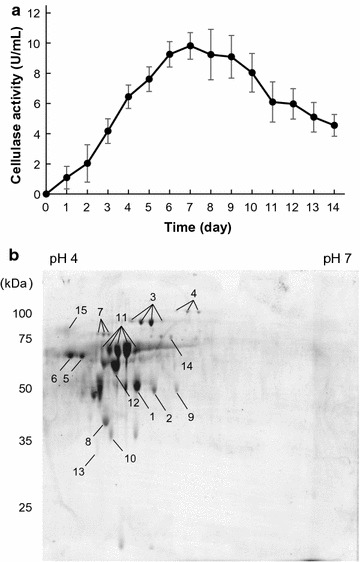
Cellulase activity and identified proteins in the culture supernatant of *Phanerochaete chrysosporium*. **a** Cellulase activity in culture supernatant from *P. chrysosporium* grown with 1.0% MCC for 0–14 days. Error bars represent the mean ± standard error of the mean of three independent experiments. **b** 2-DE analysis of extracellular proteins from *P. chrysosporium* grown with MCC as the sole carbon source for 7 days. Identified protein bands are marked by arrows with numbers (Table 1)

A time-course of cellulase activity in the culture supernatant of *P. chrysosporium* grown in Kirk medium containing MCC as the sole carbon resource was examined. Cellulase activity gradually increased, reaching a maximum of 9.82 U/mL at 7 days (Fig. 2a). Figure 2b shows the 2-DE profiles of extracellular proteins, where a total of 32 protein spots were detected, 26 of which (15 proteins) were identified via PMF and MS/MS analysis (Table 1). The existence of a signal peptide in these proteins was predicted using the SignalP program (http://www.cbs.dtu.dk/services/SignalP/; Fig. 2b and Table 1). Proteins identified in the MCC medium were categorized into three classes: (1) enzymes involved in cellulose degradation (spots 3–8 and 11–13), (2) enzymes involved in xylan degradation (spots 1, 2, 9, and 10), and (3) other proteins (spots 14 and 15) (Table 1). The protein corresponding to spot 14 was identified as glyoxal oxidase (Table 1). Among the detected proteins, the major cellulolytic enzymes were CBHI belonging to the GH7 family (Fig. 2b; spots 5, 6, and 11) and CBHII belonging to the GH6 family (Fig. 2b; spot 12)

.

**Table 1:** Proteins identified in the culture supernatant of *Phanerochaete chrysosporium*

No.^a^	Protein name	Protein ID	tpI^b^	tMW^c^	Cov^d^	IP^e^	Sig^f^
1	Acetylxylan esterase (CE1)	126075	5.9	35.6	21	2	+
2	Acetylxylan esterase (CE1)	129015	5.9	39.5	20	2	+
3	Cellobiose dehydrogenase (CDH)	11098	5.2	82.0	19	2	+
4	Cellulose-binding-β-glucosidase (GH3)	134658	5.4	85.2	23	5	+
5	Cellobiohydrolase I (GH7)	137372	5.0	58.1	26	3	+
6	Cellobiohydrolase I (GH7)	137216	4.3	53.8	17	4	+
7	CEL6 protein (GH74)	138266	4.8	77.9	16	3	+
8	Endo-glucanase II (GH5)	6458	5.6	41.8	33	3	+
9	Endo-1,4-β-xylanase I (GH10)	7045	6.5	39.5	28	2	+
10	Endo-1,4-β-xylanase II (GH11)	133788	5.7	30.4	19	1	+
11	Cellobiohydrolase I (GH7)	127029	5.0	55.0	16	3	+
12	Cellobiohydrolase II (GH6)	133052	5.0	48.4	14	3	+
13	Exo-1,4-β-glucanase (GH12)	8466	4.7	25.4	25	3	+
14	Glyoxal oxidase	11068	5.2	59.5	18	2	+
15	Putative copper radical oxidase 2	134241	4.9	78.1	14	2	+

^a^Numbers 1–15 indicate identified proteins in the MCC medium. The MASCOT scores of PMFs and MS/MS ion searches were > 77 and > 63, respectively

^b^Theoretical pI

^c^Theoretical mass

^d^Sequence coverage (%) in PMF

^e^Identified peptides by MS/MS ion search

^f^Presence (+) of a signal peptide was predicted using the SignalP program (http://www.cbs.dtu.dk/services/SignalP/)

### Decreases in CMC-solution viscosity by oxygen-radical pretreatment

**Fig. 3:**
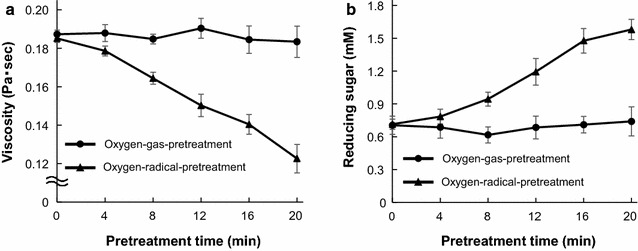
Effects of oxygen-radical pretreatment on the viscosity of CMC solutions and CMC hydrolysis. **a** Apparent viscosity of the CMC suspensions pretreated with oxygen gas and plasma 2.0% (w/v) was measured using a rheometer at a shear rate of 200 s^−1^. Values were normalized based on the effects of temperature and shearing on viscosity. **b** Pretreatment time-dependent production of reducing sugars released from oxygen-gas- (circles) or oxygen-radical-pretreated (triangles) CMC by enzymatic hydrolysis using culture supernatant was assayed using the DNS method. Error bars represent the mean ± standard error of the mean of three independent experiments

The apparent viscosity of the CMC aqueous suspensions is shown in Fig. 3a. The viscosity of the suspension treated with oxygen gas was approximately constant within experimental error, whereas the viscosity of that treated with oxygen radicals decreased significantly along with increasing treatment time. Benoit et al. [42] showed that cellulose is partially depolymerized by non-thermal atmospheric plasma (NTAP) in a dielectric barrier discharge reactor. The degree of polymerization of MCC decreases from 250 to 120 after NTAP treatment, suggesting that this treatment is capable of cleaving the β-1,4-glycosidic bond without the assistance of any catalyst or solvent [43]. Similar results were also observed using other grades of cellulose (α-cellulose, bacterial cellulose, and cotton). Although the exact mechanism of cellulose depolymerization by plasma treatment is not yet fully elucidated, plasma treatment would likely generate radicals in the gas phase, which react with glucosyl units on the surface of cellulose to form radicals that lead to bond cleavage [2]. The most favorable cleavage occurs at the β-1,4-glycoside linkage, as suggested by electron spin resonance spectroscopy analysis [44, 45]. Based on these findings, the decrease in viscosity in the CMC suspensions treated with oxygen radicals indicated that the β-1,4-glycoside linkages of the cellulose backbone were cleaved by radical pretreatment.

### Effects of oxygen-radical pretreatment on the production of reducing sugars from CMC using culture supernatant

**Fig. 4:**
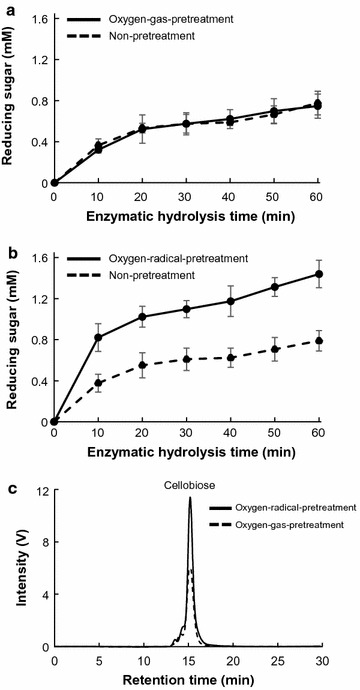
Effects of oxygen-radical pretreatment on CMC hydrolysis by cellulolytic enzymes in culture supernatant. Time-course of the release of reducing sugars from **a** oxygen-gas- or **b** oxygen-radical-pretreated CMC by enzymatic hydrolysis using culture supernatant and assayed using the DNS method. Error bars represent the mean ± standard error of the mean of three independent experiments. **c** Reaction products from pretreated CMC by enzymatic hydrolysis for 60 min were analyzed by reducing-sugar HPLC

A time-course analysis of reducing-sugar production from CMC using the culture supernatant of *P. chrysosporium* grown with MCC for 7 days following oxygen-gas and oxygen-radical pretreatment for 0, 4, 8, 12, and 20 min was performed (Fig. 3b). Reducing-sugar production in oxygen-radical-pretreated suspensions increased significantly along with increasing treatment time (Fig. 3b). The concentration of reducing sugars produced from CMC in suspensions pretreated with oxygen gas or oxygen radical for 20 min was also determined. The soluble-sugar production by the enzyme mixture in the culture supernatant obtained from *P. chrysosporium* grown with MCC for 7 days was determined using the DNS method. Oxygen-gas pretreatment did not affect reducing-sugar production by the supernatant of *P. chrysosporium* cultures (Fig. 4a). Compared with CMC suspensions with or without pretreatment with oxygen gas, the concentration of reducing sugar was significantly increased in oxygen-radical-pretreated CMC suspensions (Fig. 4b). After a 60-min reaction, the production of reducing sugars from radical-pretreated CMC reached levels 2.0-fold higher than those from oxygen-gas-pretreated and non-pretreated CMC (Fig. 4b). The soluble products from radical-pretreated CMC were analyzed by reducing-sugar HPLC, which detected cellobiose (1.5 mM) as a product from radical-pretreated CMC following enzymatic hydrolysis for 60 min (Fig. 4c). The reaction product from radical-pretreated CMC was 2.0-fold more abundant than that from oxygen-gas-pretreated CMC, indicating that radical pretreatment enhanced enzymatic hydrolysis in the culture supernatant containing various cellulases. Similar results were observed using MCC as a substrate (Additional file 1: Figure S1).

**Fig. 5:**
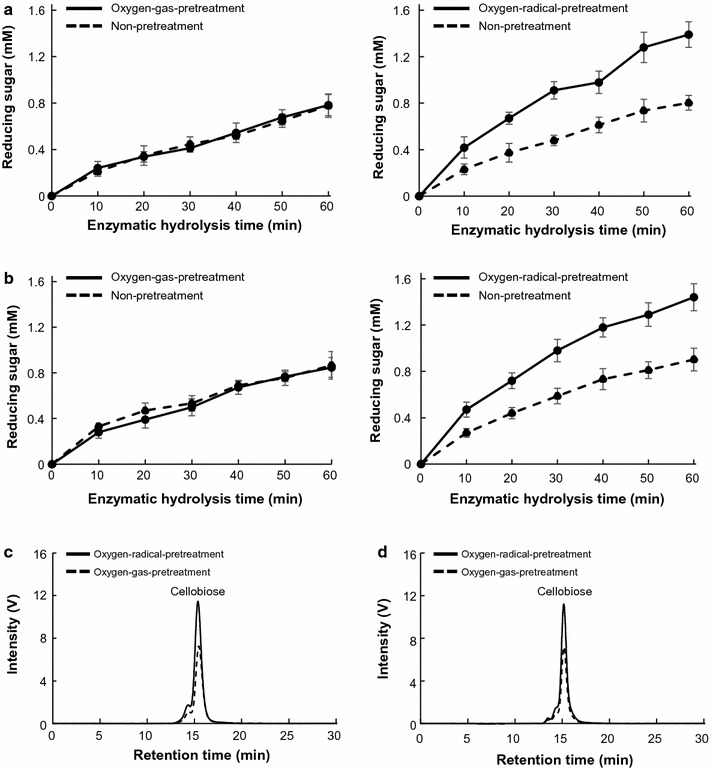
Effects of oxygen-radical pretreatment on enzymatic hydrolysis of CMC. **a**, **b** Reducing-sugar production and **c**, **d** reaction products from oxygen-radical-pretreated CMC by **a**, **c** CBHI and **b**, **d** CBHII were analyzed. Error bars represent the mean ± standard error of the mean of three independent experiments

Reducing-sugar production from oxygen-gas- and oxygen-radical-pretreated CMC suspensions digested with CBHI and CBHII was also evaluated (Fig. 5). Similar reaction product was obtained when culture supernatant was used (Fig. 5c). Reducing-sugar production from radical-pretreated CMC digested with CBHI and CBHII was 1.7- and 1.6-fold higher, respectively, than that from non-pretreated CMC, whereas oxygen-gas pretreatment did not promote the production of reducing sugars from CMC (Fig. 5). These results suggested that cleavage of the cellulose backbone into smaller chains by radical pretreatment promoted cellulose hydrolysis by the processing of reducing- and non-reducing-end cellulases. Furthermore, the increases in the rate of reducing-sugar production from radical-pretreated CMC digested with CBHI and CBHII were lower than those observed in culture supernatant (Fig. 4), suggesting that synergistic hydrolysis by cellulases capable of processing reducing- and non-reducing ends (CBHI and CBHII) and other cellulolytic enzymes, including endo-glucanases and LPMOs, in culture supernatant might have occurred.

### Effects of oxygen-radical pretreatment on the enzyme hydrolysis of wheat straw

The composition of cellulose, hemicellulose, and lignin in non-pretreated, oxygen-gas-pretreated, and oxygen-radical-pretreated wheat straw (Additional file 1: Table S1) was determined. Oxygen-gas and oxygen-radical pretreatments did not affect the composition of wheat straw, indicating that oxygen-radical pretreatment might cause the depolymerization of wheat-straw components, including cellulose, but did not convert small molecules, such as mono- and oligo-saccharides and water-soluble aromatic compounds. The previous studies reported that plasma pretreatment of wheat straw causes lignin depolymerization [2, 46, 47]. Because lignin-degradation products affect enzymatic degradation of cellulose, the washing step of plasma-pretreated wheat straw with water is important to eliminate inhibitory components [2, 46, 47]. In the present study, oxygen-gas- and oxygen-radical-pretreated wheat straw was washed with water to eliminate enzyme inhibitors according to previously described methods [47]. The products collected from washing oxygen-gas- and oxygen-radical-pretreated wheat straw were lyophilized, trimethylsilylated, and analyzed by gas chromatography MS; however, no sugars or lignin-degradation products were detected (Additional file 1: Figure S2). These results indicated that oxygen-radical pretreatment using a radical generator based on non-thermal atmospheric pressure plasma technology did not produce low-molecular-weight lignin-degradation products. A different pretreatment time might be an explanation for the differences in time scales between those (from 0.5 to 7 h) cited by Vanneste et al. and Schult-Jensen et al. [2, 46, 47] and that (20 min) mentioned in this study.

**Fig. 6:**
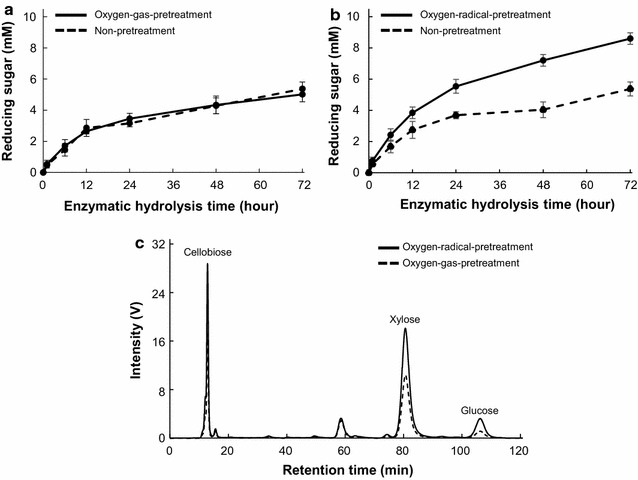
Effects of oxygen-radical pretreatment on the enzymatic hydrolysis of wheat straw. Reducing sugars released from **a** oxygen-gas- and **b** oxygen-radical-pretreated wheat straw after enzymatic hydrolysis using the supernatant from *Phanerochaete chrysosporium* cultures were assayed using the DNS method. **c** Reaction products from pretreated wheat straw by enzymatic hydrolysis for 48 h were analyzed by reducing-sugar HPLC. Data are presented as the mean ± standard deviation of three experiments

The production of reducing sugars from washed and unwashed oxygen-radical-pretreated wheat straw was determined (Additional file 1: Figure S3), with the amount of reducing sugars from both wheat-straw samples was similar following enzymatic hydrolysis for 48 h. Based on this observation, unwashed oxygen-radical-pretreated wheat straw was used for subsequent hydrolysis studies. After 1-, 6-, 12-, 24-, 48-, and 72-h reactions using culture supernatant, the amount of reducing sugars from oxygen-radical-pretreated wheat straw reached levels 1.4-, 1.4-, 1.4-, 1.5-, 1.8-, and 1.8-fold, respectively, higher than those from oxygen-gas-pretreated wheat straw (Fig. 6b). The soluble products from radical-pretreated wheat straw were analyzed by reducing-sugar HPLC, which detected cellobiose (4.7 mM) as the predominant product from radical-pretreated wheat straw following enzymatic hydrolysis for 48 h (Fig. 6c). After a 10- to 60-min hydrolytic reaction, the production of reducing sugars from radical-pretreated CMC consistently reached levels ~ 2.0-fold higher than those from oxygen-gas-pretreated CMC (Fig. 4b). Analysis of the soluble products from radical-pretreated wheat straw by HPLC (Fig. 6c) revealed glucose (0.5 mM) and xylose (2.3 mM), which were undetectable in reactions associated with CMC, after 48 h (Fig. 6c). Moreover, the conversion rate between oxygen-gas- and oxygen-radical-pretreated wheat straw gradually increased (Fig. 6b), suggesting that enhanced cellulose hydrolysis promoted by depolymerization of the cellulose backbone by oxygen-radical pretreatment might synergistically promote hemicellulose hydrolysis in culture supernatant.

An advantage of oxygen-radical pretreatment of plant biomass relative to the conventional chemical processing is the use of dry gases instead of chemicals and solvents, which eliminates the need for sample filtration/purification, chemical recovery, and waste treatment. Oxygen-radical pretreatment also eliminates the additional costs associated with the use and purchase of these chemicals. In addition, plasma discharge generated electrically might represent an attractive pretreatment process through the use of renewable energy. Compared with the conventional pretreatment methods, oxygen-radical pretreatment is currently rarely used; however, it is important to thoroughly assess the sustainable character of oxygen-radical pretreatment of biomass. For industrial use, it is also important to keep the costs associated with enzyme production low. In general, the enzyme-purification step is time-consuming and accounts for up to 80% of the total production cost of the enzymes [48]. Use of supernatant obtained from *P. chrysosporium* cultures that mainly produce cellulases that process reducing- and non-reducing ends (CBHI and CBHII) reduces the purification cost of these enzymes.

## Conclusions

In this study, the effects of oxygen-radical pretreatment on enzymatic hydrolysis of CMC and wheat straw were analyzed. Oxygen-radical pretreatment enhanced the degradation of cellulose by reducing- and non-reducing-end cellulases in supernatant from white-rot fungus *P. chrysosporium* cultures. The reaction products from oxygen-radical-pretreated CMC and wheat straw following degradation by cellulases in culture supernatant were 2.0- and 1.8-fold more abundant than those from oxygen-gas-pretreated CMC and wheat straw, respectively. Our findings suggested that oxygen-radical pretreatment of plant biomass offers great promise for further improvements in lignocellulose-deconstruction processes.

## Additional file

**Additional file 1: Table S1.** The content of cellulose, hemicellulose, and lignin in non-pretreated, oxygen-gas-pretreated, and oxygen-radical-pretreated wheat straw. **Figure S1.** Effects of oxygen-radical pretreatment on MCC hydrolysis by cellulolytic enzymes in culture supernatant. Reducing sugars released from (a) oxygen-gas- or (b) oxygen-radical-pretreated MCC by enzymatic hydrolysis using culture supernatant were assayed using the DNS method. Error bars represent the mean ± standard error of the mean of three independent experiments. **Figure S2.** Gas chromatography spectra of the washing water of oxygen-gas- and oxygen-radical-pretreated wheat straw. (a) Oxygen-gas- and (b) oxygen-radical-pretreated wheat-straw samples were extracted with water to wash out enzyme inhibitors. Each treatment sample was washed with 25°C Milli-Q water, followed lyophilization, trimethylsilylation, and analysis of the liquid fraction by gas chromatography. **Figure S3.** Reducing-sugar production from washed and unwashed oxygen-radical-pretreated wheat straw. Reducing sugars released from washed and unwashed oxygen-radical-pretreated wheat straw after enzymatic hydrolysis using the supernatant from *Phanerochaete chrysosporium* cultures were assayed using the DNS method. Data are presented as the mean ± standard deviation of three experiments.

## Abbreviations

CBH: cellobiohydrolase
CMC: carboxymethyl cellulose
GH: glycoside hydrolase
DNS: dinitrosalicylic acid
LPMO: lytic polysaccharide monooxygenase
MCC: microcrystalline cellulose
NTAP: non-thermal atmospheric plasma
HPLC: high-performance liquid chromatography
MS: mass spectrometry
PMF: peptide mass fingerprinting
2-DE: two-dimensional gel electrophoresis
VUVAS: vacuum ultraviolet absorption spectroscopy
