# Supplementary material for: Oxygen-radical pretreatment promotes cellulose degradation by cellulolytic enzymes
Source: Biotechnol Biofuels. 2017 Dec 4;10:290. doi: 10.1186/s13068-017-0979-6 (PMC5713004; doi:10.1186/s13068-017-0979-6)
Supplement: Supplementary file 1 — Additional file 1: Table S1. The content of cellulose, hemicellulose, and lignin in non-pretreated, oxygen-gas-pretreated, and oxygen-radical-pretreated wheat straw. Figure S1. Effects of oxygen-radical pretreatment on MCC hydrolysis by cellulolytic enzymes in culture supernatant. Reducing sugars released from (a) oxygen-gas- or (b) oxygen-radical-pretreated MCC by enzymatic hydrolysis using culture supernatant were assayed using the DNS method. Error bars represent the mean ± standard error of the mean of three independent experiments. Figure S2. Gas chromatography spectra of the washing water of oxygen-gas- and oxygen-radical-pretreated wheat straw. (a) Oxygen-gas- and (b) oxygen-radical-pretreated wheat-straw samples were extracted with water to wash out enzyme inhibitors. Each treatment sample was washed with 25°C Milli-Q water, followed lyophilization, trimethylsilylation, and analysis of the liquid fraction by gas chromatography. Figure S3. Reducing-sugar production from washed and unwashed oxygen-radical-pretreated wheat straw. Reducing sugars released from washed and unwashed oxygen-radical-pretreated wheat straw after enzymatic hydrolysis using the supernatant from Phanerochaete chrysosporium cultures were assayed using the DNS method. Data are presented as the mean ± standard deviation of three experiments. [file 13068_2017_979_MOESM1_ESM.docx]

**Additional file**

**Oxygen-radical pretreatment promotes cellulose degradation by cellulolytic enzymes**

Kiyota Sakai^1^, Saki Kojiya^1^, Junya Kamijo^1^, Yuta Tanaka^2^, Kenta Tanaka^1^, Masahiro Maebayashi^1^, Jun-Seok Oh^2^, Masafumi Ito^2^, Masaru Hori^3^, Motoyuki Shimizu^1^*****, Masashi Kato^1^

^1^ Faculty of Agriculture, Meijo University, Nagoya, Aichi 468-8502, Japan

^2^ Faculty of Science and Technology, Meijo University, Nagoya, Aichi 468-8502, Japan

^3^ Institute of Innovation for Future Society, Nagoya University, Nagoya, Aichi 464-8603, Japan

***** Corresponding author.

Tel/Fax: +81-52-838-2445

E-mail: moshimi@meijo-u.ac.jp (M. Shimizu).

**Table S1. The content of cellulose, hemicellulose and lignin in non-pretreated, oxygen-gas-pretreated, and oxygen-radical-pretreated wheat straw.**

| Pretreatment | Composition (%)^a^ | | | Weight loss (%)^b^ |
| --- | --- | --- | --- | --- |
|  | Cellulose | Hemicellulose | Lignin |  |
| None | 35.9 ± 4.5 | 24.4 ± 2.7 | 23.5 ± 1.2 | 1.4 ± 0.5 |
| Oxygen gas | 33.4 ± 2.7 | 25.6 ± 3.5 | 23.4 ± 0.8 | 0.8 ± 0.3 |
| Oxygen radical | 34.7 ± 3.4 | 23.1 ± 2.0 | 23.8 ± 0.7 | 1.2 ± 0.7 |

^a^ Based on dry matter.

^b^ Referring to the initial dry weights.

The content of cellulose, hemicellulose, and lignin in the oxygen-gas- and oxygen-radical-pretreated wheat straw was determined according to previous methods. Data are presented as the mean ± standard deviation of three experiments.

Fig. S1
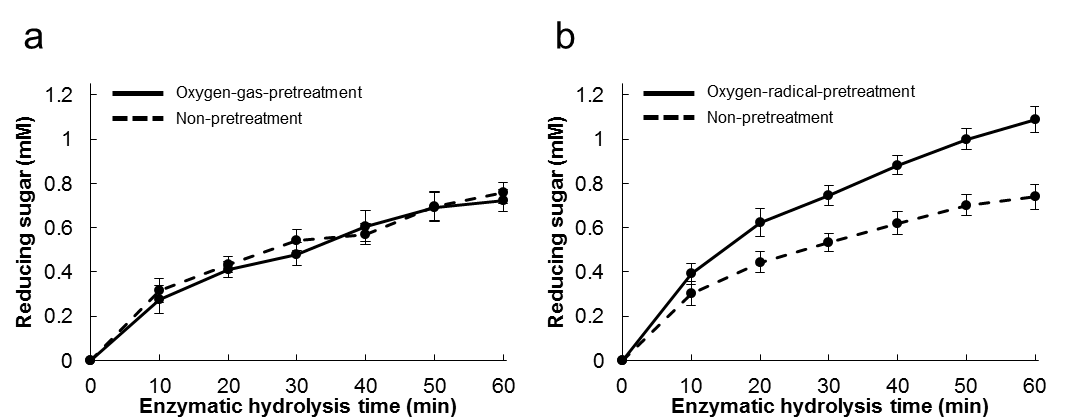


**Fig. S1. Effects of oxygen-radical pretreatment on MCC hydrolysis by cellulolytic enzymes in culture supernatant.** Reducing sugars released from (a) oxygen-gas- or (b) oxygen-radical-pretreated MCC by enzymatic hydrolysis using culture supernatant were assayed using the DNS method. Error bars represent the mean ± standard error of the mean of three independent experiments.

Fig. S2

**
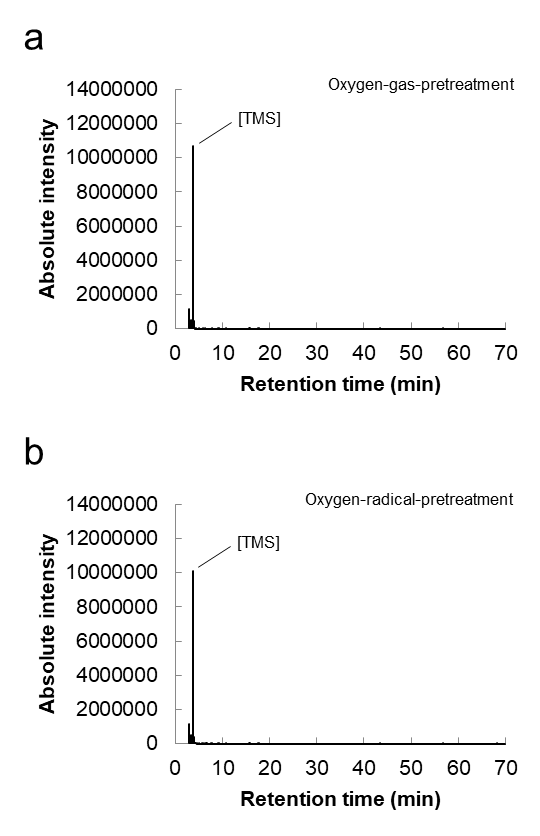
**

**Figure S2. Gas chromatography spectra of the washing water of oxygen-gas- and oxygen-radical-pretreated wheat straw.** (a) Oxygen-gas- and (b) oxygen-radical-pretreated wheat-straw samples were extracted with water to wash out enzyme inhibitors. Each treatment sample was washed with 25°C Milli-Q water, followed lyophilization, trimethylsilylation, and analysis of the liquid fraction by gas chromatography.

Fig. S3


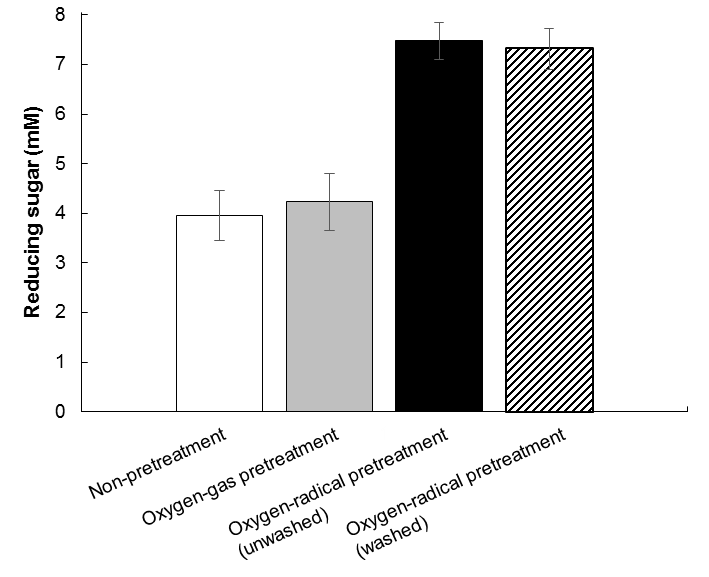


**Figure S3 Reducing-sugar production from washed and unwashed oxygen-radical-pretreated wheat straw.** Reducing sugars released from washed and unwashed oxygen-radical-pretreated wheat straw after enzymatic hydrolysis using the supernatant from *Phanerochaete chrysosporium* cultures were assayed using the DNS method. Data are presented as the mean ± standard deviation of three experiments.
